# Gut-liver axis dysregulation and restoration in decompensated hepatitis B cirrhosis: rationale and research progress of fecal microbiota transplantation

**DOI:** 10.3389/fmicb.2026.1949414

**Published:** 2026-10-14

**Authors:** Shujuan Gao, Jie Zhuang, Hui Cheng, Kai Hu, Liwei Zhuang, Yong Pan

**Affiliations:** 1 Department of Gastroenterology, Huaihai Hospital Affiliated to Xuzhou Medical University, Xuzhou, China; 2 Department of Hepatology, Xuzhou Infectious Disease Hospital, Xuzhou, China

**Keywords:** cirrhosis, decompensated, fecal microbiota transplantation, gut microbiota, gut-liver axis, hepatitis B virus

## Abstract

Decompensated hepatitis B virus (HBV) related cirrhosis is associated with high mortality and a wide range of complications. Current antiviral and symptomatic treatments fail to reverse core pathological alterations, including disruption of the gut microbial ecosystem. Gut-liver axis imbalance represents a central hub in disease progression. Gut dysbiosis compromises the intestinal mucosal barrier, leading to endotoxin translocation into the bloodstream, which in turn exacerbates hepatic inflammation and fibrosis, thereby establishing a vicious cycle. This review synthesizes the key mechanisms, clinical efficacy, administration routes, and safety profile of fecal microbiota transplantation (FMT) in this disease. It also identifies current technical bottlenecks and research gaps in FMT application and discusses future directions toward precision based and standardized clinical translation of this therapeutic approach.

## 1 Introduction

Chronic hepatitis B virus (HBV) infection remains a major component of the global liver disease burden, particularly in Asia and Africa (Cui et al., 2026). Although nucleos(t)ide analogs and interferon therapies can effectively suppress viral replication and delay disease progression, a considerable proportion of patients still develop cirrhosis under conditions of prolonged viral carriage or inadequate viral suppression (Dusheiko et al., 2023; Tang et al., 2025; European Association for the Study of the Liver, 2025). Decompensation represents a turning point in the natural history of cirrhosis, marking the onset of severe hepatic reserve depletion and systemic pathophysiological disturbances. Clinically, it manifests as hepatic encephalopathy (HE), spontaneous bacterial peritonitis (SBP), esophagogastric variceal bleeding, or jaundice (Fattovich et al., 1995; Ginès et al., 2021; Villanueva et al., 2021; Villanueva et al., 2025). Epidemiological data indicate that the annual mortality rate of patients with decompensated HBV-related cirrhosis is 5–10 times higher than that of patients with compensated cirrhosis, with a 5-year survival rate below 30% (Fattovich et al., 2002; Hui et al., 2002). The leading causes of death are not liver failure alone but its serious complications: recurrent SBP can precipitate sepsis and acute-on-chronic liver failure (ACLF); HE severely impairs quality of life and increases the risk of accidental death; and portal hypertensive events further shorten survival (Huelin et al., 2017; Ginès et al., 2018; Kaplan et al., 2024). Current treatment strategies include antiviral therapy to reduce viral load, diuretics for ascites control, lactulose or rifaximin to prevent HE, antibiotics for SBP, and endoscopic therapy for varices. However, these interventions are mostly symptomatic or target specific complications, and they are unable to reverse the overall disease course (Peng et al., 2012; European Association for the Study of the Liver, 2025). Liver transplantation, although potentially curative, is accessible to only a very small subset of patients due to organ shortage, high costs, and risks associated with immunosuppression. Therefore, exploring novel therapeutic strategies that intervene upstream in the pathophysiology of the decompensation process in cirrhosis is of significant clinical importance.

In recent years, the concept of the “gut-liver axis” has provided a entirely new perspective on the pathogenesis of decompensated cirrhosis (Hsu and Schnabl, 2023; Pabst et al., 2023). The liver and intestine are anatomically and functionally closely connected via the portal venous system: gut-derived nutrients, bacteria, and their metabolites enter the liver through the portal vein, where they are cleared or metabolized by Kupffer cells, hepatocytes, and liver sinusoidal endothelial cells (Gola et al., 2021; Guilliams et al., 2022). Under healthy conditions, the gut microbiota is in a state of homeostasis, and an intact intestinal epithelial barrier strictly restricts the translocation of live bacteria and pathogen-associated molecular patterns (PAMPs) into the circulation (Martel et al., 2022). Once this barrier is compromised, large quantities of intestinal bacteria or their products translocate into the portal and systemic circulation, thereby inducing hepatic and systemic inflammation and injury (Hasa et al., 2022). The role of microbial metabolites is particularly critical. Lipopolysaccharide (LPS), a component of the Gram-negative bacterial cell wall, activates hepatic innate immunity via Toll-like receptor 4 (TLR4), promoting the release of inflammatory cytokines and the activation of hepatic stellate cells, thereby accelerating fibrosis progression (Granowitz et al., 1991; Poltorak et al., 1998; Tang et al., 2026). Short-chain fatty acids (SCFAs), produced mainly by Firmicutes and Bacteroidetes, help maintain the intestinal barrier, regulate Treg cell differentiation, and exert anti-inflammatory effects; their production is significantly reduced in cirrhosis (Chen et al., 2011; Park et al., 2015; Chu et al., 2019). The enterohepatic circulation of bile acids (BAs) is regulated by the gut microbiota, as the conversion of primary BAs to secondary BAs depends on 7α/β-dehydroxylation by specific anaerobic bacteria. Patients with cirrhosis exhibit an abnormal bile acid profile, and the reduction in secondary BAs further weakens intestinal immune defense (Kakiyama et al., 2013; Ridlon et al., 2013; Perino et al., 2021; Perino and Schoonjans, 2022). Thus, the proper functioning of the gut-liver axis depends on the synergistic stability of gut microbiota structure and function, intestinal barrier integrity, and hepatic immune-metabolic capacity. Dysregulation of any one of these components can initiate a vicious cycle.

In patients with decompensated HBV-related cirrhosis, the specific manifestations of gut-liver axis dysregulation have been substantiated by multi-omics studies. These include a marked decline in gut microbial diversity, reduced abundance of commensal bacteria, particularly Firmicutes and Bacteroidetes, and overgrowth of potentially pathogenic bacteria such as Enterobacteriaceae, Streptococcaceae, and Staphylococcaceae. In some patients, even ectopic colonization resembling respiratory tract- or skin-like microbiota has been observed (Qin et al., 2014; Bajaj et al., 2017a, 2017b; Jin et al., 2026). The direct consequences of microbial dysbiosis include reduced secretion of antimicrobial peptides, thinning of the mucus layer, and downregulation of tight junction proteins, leading to increased intestinal permeability, i.e., the “leaky gut” (Teltschik et al., 2012; Acharya and Bajaj, 2017). This is followed by translocation of bacterial products and even live bacteria, manifested by elevated plasma levels of LPS, lipoteichoic acid, and other markers, resulting in endotoxemia (Clària et al., 2016; Arroyo et al., 2021; Albillos et al., 2022). Concurrently, cirrhosis-associated portal hypertension leads to intestinal congestion, reduced motility, and bacterial overgrowth, which further exacerbates dysbiosis and intestinal barrier disruption. Together, these factors establish a vicious cycle in which dysbiosis promotes barrier disruption, which in turn fuels endotoxemia and liver injury, thereby aggravating portal hypertension and perpetuating further dysbiosis (Bosch and García-Pagán, 2000). Moreover, HBV infection itself may aggravate this process through direct hepatocyte injury and indirect effects of viral proteins on gut immunity (Chou et al., 2015; Tsai et al., 2018). Hepatitis B e antigen (HBeAg) primarily fosters an immunosuppressive environment by inducing T cell tolerance, driving a Th2-dominant response, suppressing TLR/IL-18 signaling, and expanding Tregs (Milich et al., 1997; Chen et al., 2004; Jegaskanda et al., 2014; Tang et al., 2020). In HBV-related cirrhosis, no studies have yet reported a role for HBx in regulating intestinal immunity. In inflammatory bowel disease, however, Hepatitis B virus X protein (HBx) promotes a pro-inflammatory microenvironment by disrupting the intestinal epithelial barrier, inhibiting RIG-I-MAVS innate immune signaling, and altering the composition of intestinal immune cells (Massimino et al., 2023). Together, these mechanisms constitute a strategy by which HBV manipulates host immunity, including intestinal immunity, to support its persistent infection. Although the mechanisms underlying cirrhosis of differing etiologies converge at the levels of microbiota, barrier, and immunity, all are centered on a shared framework of portal hypertension driven “gut-liver axis” disruption. Etiology and clinical context do not alter the fundamental nature of the three pathways, namely dysbiosis, barrier compromise, and immune dysfunction; rather, they shape their phenotype and severity atop these common mechanisms. HBV cirrhosis is characterized immunologically by T cell exhaustion driven by persistent antigen exposure, whereas alcohol related cirrhosis features myeloid cell expansion mediated immunosuppression, and alcohol’s direct toxicity to the intestinal epithelium, superimposed on portal hypertension, renders barrier injury and microbial destabilization more profound (Gao et al., 2019; Gu et al., 2024; Yao et al., 2024; Raya Tonetti et al., 2024). The distinguishing feature of HBV cirrhosis lies in the accessibility of antiviral therapy: nucleos(t)ide analogues can partially restore microbial diversity, indicating that its dysbiosis is to a considerable extent a reversible consequence of viral replication (Li et al., 2022; Shen et al., 2023); alcohol, by contrast, causes intestinal epithelial injury, and when superimposed on portal hypertension, produces more severe barrier damage (Nicoletti et al., 2019). Even when the virus is suppressed, microbiota immune dysregulation at the cirrhotic stage can become partially “autonomous,” with hepatocellular carcinoma (HCC) risk persisting (Wang et al., 2015). Thus, gut-liver axis dysregulation in decompensated HBV-related cirrhosis is not a static pathological state, but rather a dynamically evolving, self-amplifying process involving multiple interacting targets.

Based on the pathophysiological logic outlined above, restoring gut-liver axis homeostasis represents a rational strategy to intervene in the course of decompensated cirrhosis. Among various microbiota-targeted interventions, probiotics have shown limited efficacy with substantial interindividual variability, whereas antibiotics, although capable of suppressing certain pathogenic bacteria, fail to restore microbial diversity (Bass et al., 2010; Lunia et al., 2014; Fernández et al., 2016). Fecal microbiota transplantation (FMT), by virtue of its ability to deliver a complete, functionally active, and ecologically stable microbial community in a single intervention, holds promise for achieving such restoration. Preliminary clinical studies have indicated that FMT can reduce blood ammonia levels, decrease recurrence of HE, improve Child-Pugh and MELD scores, and lower the incidence of SBP and other infectious events in patients with decompensated cirrhosis (Bajaj et al., 2017a, 2017b; Bajaj et al., 2019). However, these studies are limited by small sample sizes, high donor heterogeneity, short follow-up periods, and inconsistent endpoint measures. Whether FMT can achieve safe and durable gut-liver axis restoration specifically in decompensated HBV-related cirrhosis therefore remains to be established by high-quality evidence-based medicine.

This review starts by examining the key steps in the disruption of the gut–liver axis, and then provides a comprehensive overview of the molecular and ecological basis for FMT-mediated restoration of this axis. We summarise available preclinical and clinical evidence, discuss current challenges such as donor standardisation, engraftment efficiency, safety, and combination regimens, and conclude with future perspectives.

## 2 Pathological characteristics of gut-liver axis dysregulation in decompensated HBV-related cirrhosis

The pathological evolution of decompensated HBV-related cirrhosis is not a linear deterioration of a single organ but rather a comprehensive collapse of the bidirectional crosstalk between the intestinal microbial ecosystem and the liver. This state of “gut-liver axis dysregulation” is essentially a multistep, positive-feedback-driven pathological cascade: originating from primary disruption of the microbial community structure, it sequentially triggers mechanical and immunological failure of the intestinal barrier, pathological translocation of bacterial products, extensive remodeling of the host metabolic network, and ultimately leads to destabilization of both local hepatic and systemic immune responses (Figure 1).

**Figure 1:**
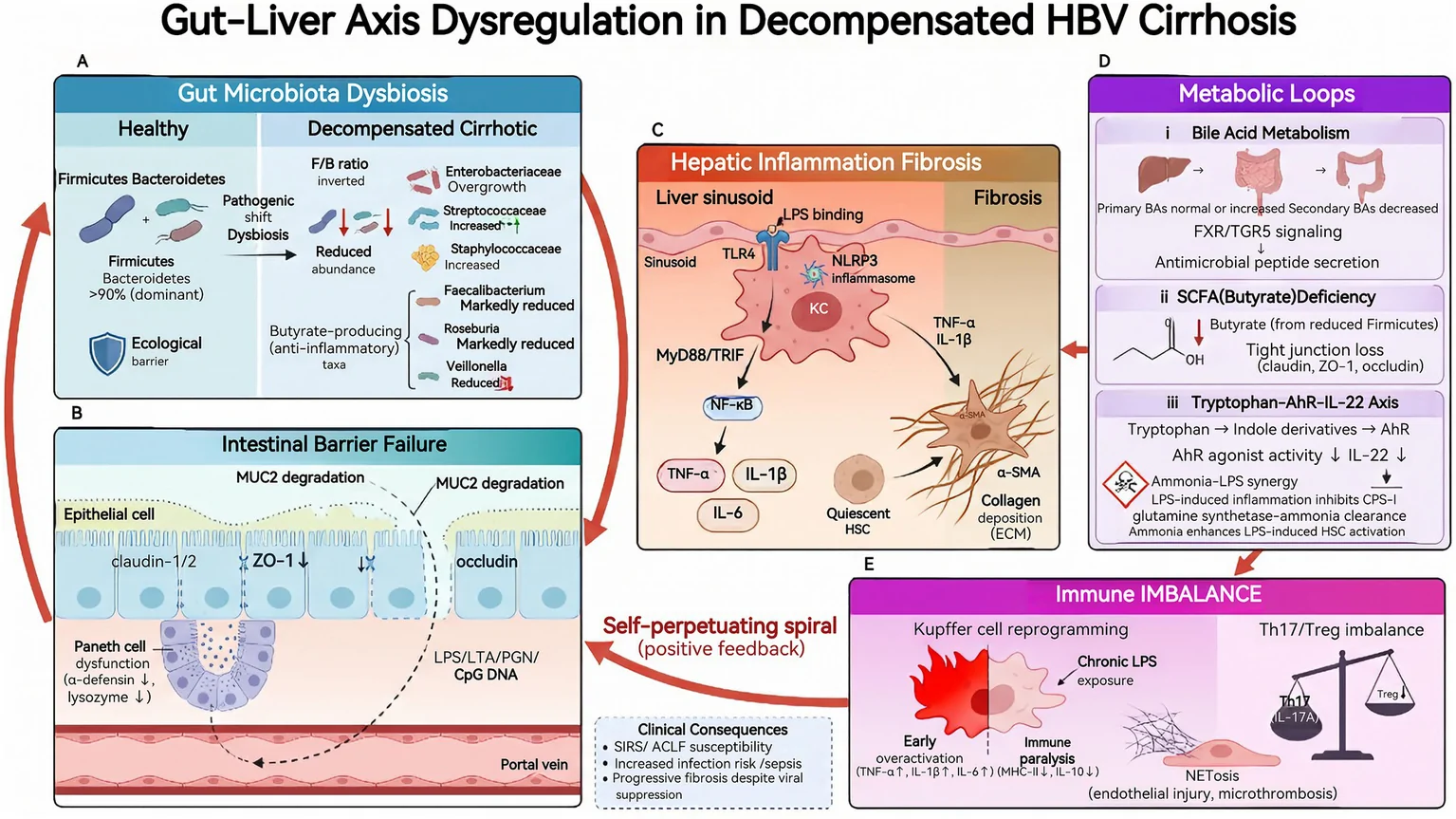
Gut-liver axis dysregulation in decompensated HBV cirrhosis. This schematic delineates the multicomponent, self-perpetuating pathological cascade underlying gut-liver axis dysregulation in decompensated hepatitis B virus (HBV)-related cirrhosis. The diagram is organized into five interconnected modules arranged in a clockwise loop, with curved red arrows denoting positive feedback that renders the system self-sustaining. **(A)** In healthy individuals, Firmicutes and Bacteroidetes together constitute >90% of the total gut microbiota, forming an ecological barrier. In decompensated HBV cirrhosis, the Firmicutes/Bacteroidetes ratio becomes inverted, accompanied by marked reductions in butyrate-producing taxa and concurrent overgrowth of potentially pathogenic facultative anaerobes, including Enterobacteriaceae, Streptococcaceae, and Staphylococcaceae. **(B)** Downregulation of tight junction proteins, degradation of the mucus layer, and Paneth cell dysfunction compromise intestinal barrier integrity. This “leaky gut” permits translocation of bacterial products, including LPS, LTA, PGN, and CpG DNA, across the epithelium into the portal circulation. **(C)** Within the liver sinusoids, translocated PAMPs activate Kupffer cells via TLR4 and the NLRP3 inflammasome. Downstream signaling through the MyD88/TRIF pathway leads to NF-κB activation and the release of proinflammatory cytokines. These cytokines drive HSC activation, toward an *α*-SMA positive myofibroblast phenotype, which deposits ECM and promotes progressive liver fibrosis. **(D)** Three interconnected metabolic disturbances further amplify gut–liver axis dysfunction: (i) secondary bile acid deficiency with impaired FXR/TGR5 signaling, reducing antimicrobial peptide secretion; (ii) SCFAs deficiency due to reduced butyrate-producing Firmicutes, compromising tight junction integrity; and (iii) suppression of the tryptophan- AhR–IL-22 axis, which impairs intestinal stem cell proliferation and epithelial repair. Additionally, ammonia and LPS exhibit synergistic hepatotoxicity: LPS-induced inflammation inhibits glutamine synthetase and CPS-I, reducing ammonia clearance, while ammonia itself enhances LPS-driven HSC activation. **(E)** Chronic LPS exposure reprograms Kupffer cells from an early hyperactivated state toward an immune-paralyzed phenotype characterized by reduced major MHC-II expression and increased IL-10 production. Simultaneously, the Th17/Treg cell balance shifts toward Th17 dominance, and excessive NETosis promotes endothelial injury and microthrombosis. This paradoxical state, proinflammatory yet immunosuppressed, explains the concurrent susceptibility to SIRS/ACLF and heightened risk of sepsis following minor infections. (ACLF, acute-on-chronic liver failure; AhR, aryl hydrocarbon receptor; BA, bile acid; CPS-I, carbamoyl phosphate synthetase I; ECM, extracellular matrix; FXR, farnesoid X receptor; HSC, hepatic stellate cell; IL, interleukin; LPS, lipopolysaccharide; LTA, lipoteichoic acid; MHC-II, major histocompatibility complex class II; NET, neutrophil extracellular trap; NF-κB, nuclear factor kappa B; NLRP3, NOD-like receptor family pyrin domain containing 3; PAMP, pathogen-associated molecular pattern; PGN, peptidoglycan; SCFA, short-chain fatty acid; SIRS, systemic inflammatory response syndrome; Th17, T helper 17 cell; TLR4, Toll-like receptor 4; Treg, regulatory T cell; α-SMA, alpha-smooth muscle actin).

### 2.1 Dysbiosis of intestinal microecology

In healthy individuals, the gut microbiota is dominated by Firmicutes and Bacteroidetes, which together account for more than 90% of the total microbial population. Through synergistic metabolism of dietary fiber, competitive inhibition of potentially pathogenic bacteria, and maintenance of an anaerobic intestinal environment, these commensals establish an “ecological barrier” (Eckburg et al., 2005; Arumugam et al., 2011). However, in patients with decompensated HBV-related cirrhosis, metagenomic studies have consistently revealed a marked reduction in microbial diversity, with both Shannon and Simpson indices significantly lower than those observed in healthy controls and patients with compensated cirrhosis (Shao et al., 2018). More critically, the compositional structure of the microbiota undergoes a “pathogenic shift”: the ratio of Bacteroidetes to Firmicute*s* becomes inverted, with notable decreases in butyrate-producing and antiinflammatory taxa suc-h as *Faecalibacterium*, *Roseburia*, and *Veillonella*, accompanied by overgrowth of facultative anaerobes represented by Enterobacteriaceae, as well as Streptococcaceae and Staphylococcaceae (Bajaj et al., 2014; Shao et al., 2018). This change is not stochastic but has clear functional implications–expansion of Enterobacteriaceae directly increases the intraluminal load of LPS; certain species of *Streptococcus* can exacerbate liver injury through surface protein-mediated interactions with hepatocytes; and overgrowth of *Staphylococcus* is closely associated with an increased risk of catheter-related infections and bacteremia (Bajaj et al., 2014). HBV infection itself, through long-range regulation of intestinal mucosal immunity by viral proteins and alterations in the selective pressure on gut microbiota resulting from reduced bile secretion in the setting of chronic liver disease, may accelerate the above pathogenic shift (Tsai et al., 2018).

### 2.2 Disruption of intestinal barrier function

The next step following microbial dysbiosis is the disruption of the mechanical and functional integrity of the intestinal barrier. The intestinal barrier is composed of the physical barrier, which includes intestinal epithelial cells and their tight junctions; the chemical barrier, including the mucus layer, antimicrobial peptides, and secretory immunoglobulin A; and the immune barrier, namely the gut associated lymphoid tissue. Its normal function is highly dependent on positive regulatory signals derived from the commensal microbiota (Johansson et al., 2008; Vaishnava et al., 2011; Dupont et al., 2014; Jakobsson et al., 2015; Mu et al., 2017; Bunker and Bendelac, 2018). Following the dysbiosis described above, butyrate producing Firmicutes are reduced, leading to decreased concentrations of butyrate, a tight junction inducing molecule. Meanwhile, overgrown Enterobacteriaceae secrete proteases that degrade mucin MUC2, thereby weakening the protective function of the mucus layer (Peng et al., 2009). At the molecular level, the key event is the downregulation of tight junction protein expression. Multiple studies of intestinal mucosal biopsies from patients with cirrhosis have confirmed that the core proteins constituting transmembrane tight junctions, namely claudin-1 and claudin-2, as well as the membrane associated scaffolding proteins ZO-1 and occludin, are significantly reduced at both transcriptional and protein levels (Sakisaka et al., 2001; Yang et al., 2008). This change converts the normally selectively permeable intestinal epithelial barrier into a “leaky gut,” allowing bacterial products with molecular weights below 500 Daltons, or even live bacteria, to pass through the paracellular route into the lamina propria.

Concurrently, Paneth cells located at the base of intestinal crypts are the primary cell type secreting antimicrobial peptides, such as *α*-defensins, lysozyme, and RegIIIγ. Their functional status directly determines the sterilizing capacity around the crypts. In the setting of decompensated cirrhosis, endotoxemia itself can induce Paneth cell degranulation and even apoptosis via TLR4 signaling. At the same time, intestinal mucosal ischemia and hypoxia resulting from portal hypertension further exacerbate their dysfunction (Gandoura et al., 2013). The consequence of impaired Paneth cell function is a loss of antimicrobial defense around the crypts. This allows small numbers of bacteria that have breached the mucus layer to colonize the crypts and continuously release antigens, thereby establishing a state of persistent low grade chronic inflammation (Teltschik et al., 2012).

The direct consequence of intestinal barrier disruption is the translocation of bacterial products and even live bacteria across the intestinal wall into the portal venous system. To date, bacterial products stably detected in the plasma of patients with decompensated HBV-related cirrhosis include, but are not limited to, LPS, which is a component of the Gram negative bacterial cell wall; lipoteichoic acid (LTA) and peptidoglycan (PGN) from Gram positive bacteria; bacterial CpG DNA; and bacterial flagellin. After these PAMPs enter the hepatic sinusoids via the portal vein, the first cells they encounter are the resident macrophages within the sinusoidal lumen, known as Kupffer cells. Kupffer cells highly express multiple pattern recognition receptors, including TLR4, TLR2, and TLR9, among which the binding of LPS to TLR4 has the greatest pathological significance (Albillos et al., 2022; Tranah et al., 2022). Upon activation of TLR4, downstream signaling pathways, both the myeloid differentiation factor 88 (MyD88) dependent pathway and the TIR-domain-containing adaptor-inducing interferon-β (TRIF) dependent pathway, ultimately activate the transcription factors nuclear factor kappa B (NF-κB) and interferon regulatory factor 3 (IRF3). This induces the transcription and release of large quantities of proinflammatory cytokines, including tumor necrosis factor alpha (TNF-α), interleukin 1 beta (IL-1β), and IL-6, as well as chemokines (Gandoura et al., 2013). Simultaneously, LPS-TLR4 signaling can also activate the NLRP3 inflammasome, promoting caspase-1 mediated maturation and secretion of IL-1β and IL-18, thereby further amplifying the inflammatory cascade (Wree et al., 2014; Trebicka et al., 2019; Liu et al., 2020). These cytokines not only directly damage hepatocytes but, more critically, activate hepatic stellate cells (HSCs). Under the combined action of TNF-α, IL-1β, and oxidative stress products, HSCs transform from quiescent lipid storing cells into a myofibroblast like phenotype expressing alpha smooth muscle actin (α-SMA). They synthesize large amounts of extracellular matrix and drive the continuous progression of liver fibrosis (Friedman, 2008; Mederacke et al., 2013; Wallace et al., 2015; Tao et al., 2019). Of note, in decompensated cirrhosis, this process has entered a “self sustaining” state. Even if the initial pathogenic stimulus, such as HBV replication, is controlled by antiviral therapy, persistent LPS translocation can maintain HSC activation. This explains the clinical conundrum of progressive liver fibrosis in many patients with well controlled viral replication.

### 2.3 Metabolic abnormalities of the gut microbiota

Beyond the immuno inflammatory framework described above, microbial metabolic abnormalities constitute another independent and critical pathological dimension of gut-liver axis dysregulation. BAs metabolism is a representative example. Primary BAs, synthesized in the liver, are excreted with bile into the intestine. Approximately 95% are reabsorbed at the terminal ileum, and the remaining 5% enter the colon. There, they are converted into secondary BAs by specific anaerobic bacteria, mainly *Clostridium* clusters XIVa and IV, *Bacteroides*, and *Eubacterium*, through 7α/β dehydroxylation (Ridlon et al., 2006; Chiang, 2009; Jia et al., 2018; Lau et al., 2025). Secondary BAs are not only essential emulsifiers for lipid absorption but also important signaling molecules. By activating the nuclear receptor farnesoid X receptor (FXR) and the membrane receptor TGR5, they induce intestinal epithelial cells to secrete antimicrobial peptides, maintain tight junction integrity, and inhibit LPS induced inflammatory responses (Lefebvre et al., 2009; Wang et al., 2011; Gadaleta et al., 2011). In patients with decompensated HBV-related cirrhosis, secondary BAs concentrations in feces and plasma are significantly reduced, and the ratio of primary to secondary BAs is elevated, reflecting loss of the dehydroxylation function of the microbiota (Kakiyama et al., 2013). This change, in turn, further weakens the intestinal defense against bacterial translocation, forming a closed loop: BAs dysregulation leads to barrier dysfunction, which allows more LPS entry and aggravated liver injury, which in turn reduces bile secretion and worsens the BAs abnormality.

The deficiency of SCFAs is equally critical. A reduction in commensal bacteria that produce SCFAs (particularly butyrate) leads to decreased colonic butyrate concentrations. Butyrate serves as both an energy substrate for intestinal epithelial cells and a histone deacetylase inhibitor; its deficiency results in downregulated expression of tight junction proteins, impaired autophagy in intestinal epithelial cells, and suppressed regulatory T cell (Treg) differentiation (Peng et al., 2009; Wang et al., 2012; Qiao et al., 2015; Feng et al., 2018). In addition, tryptophan metabolic disturbance has gained attention in recent years. The gut microbiota metabolizes dietary tryptophan into indole and its derivatives, which are endogenous ligands of the aryl hydrocarbon receptor (AhR). AhR activation is essential for maintaining the function of intraepithelial lymphocytes (IELs) and group 3 innate lymphoid cells (ILC3s). In patients with decompensated cirrhosis, fecal AhR agonist activity is significantly reduced, leading to decreased secretion of IL-22, a key cytokine that maintains intestinal stem cell proliferation and antimicrobial peptide production (Fujigaki et al., 2001; Laurans et al., 2018). Gut derived ammonia production plays a central role in hyperammonemia in cirrhosis. Furthermore, there is a synergistic hepatotoxic effect between LPS and ammonia. LPS induced inflammation inhibits the activity of key enzymes for ammonia metabolism in hepatocytes, including glutamine synthetase and carbamoyl phosphate synthetase I (CPS-I), thereby reducing ammonia clearance capacity. Conversely, ammonia itself can enhance LPS induced stellate cell activation, forming an “ammonia-LPS synergistic toxicity axis” (Frieg et al., 2021; Gallego-Durán et al., 2024; Ziki and Colnot, 2024). LPS induced inflammation inhibits the activity of key enzymes for ammonia metabolism in hepatocytes, including glutamine synthetase (GS) and carbamoyl phosphate synthetase I (CPS-I), thereby reducing ammonia clearance capacity. Conversely, ammonia itself can enhance LPS induced stellate cell activation, forming an “ammonia-LPS synergistic toxicity axis” (Frieg et al., 2021; Gallego-Durán et al., 2024; Ziki and Colnot, 2024). LPS inhibits CPS-I via protein efflux and inactivates GS via tyrosine nitration, leading to ammonia accumulation through these dual pathways (Görg et al., 2005; Unuma et al., 2013). The accumulated ammonia acts on HSCs, inducing ROS production; ROS, in turn, pre-activates NF-κB and may upregulate TLR4 expression (Jalan et al., 2016; Lu et al., 2017). Upon subsequent LPS stimulation, these ammonia-“sensitized” HSCs exhibit stronger NF-κB activation and a lower apoptosis rate, thereby amplifying pro-fibrotic signaling (Elsharkawy et al., 1999; Lang et al., 2000).

### 2.4 Dysregulation of immune homeostasis

Host immune dysregulation runs through all of the above pathological links, serving both as a consequence of gut-liver axis dysregulation and as a factor that exacerbates it. Kupffer cells lie at the center of this dysregulation. Upon long term exposure to high levels of LPS, Kupffer cells are not always in a hyperactivated state but instead undergo a unique “reprogramming” process. An early phase of overactivation leads to massive release of proinflammatory cytokines (Tacke, 2017; Peiseler et al., 2023; Horn and Tacke, 2024). With continuous LPS stimulation, Kupffer cells gradually enter a state of so called “endotoxin tolerance” or “immune paralysis,” characterized by decreased antigen presenting capacity, reduced major histocompatibility complex class II (MHC-II) expression, and relatively increased production of antiinflammatory cytokines such as IL-10 (Kurachi et al., 2003; Seki et al., 2007). This state may be protective by limiting excessive inflammation, but the cost is significantly compromised host defense against subsequent infections. This explains why patients with decompensated cirrhosis are prone not only to systemic inflammatory response syndrome (SIRS) and ACLF, but also to rapid progression to sepsis following even minor infections. Furthermore, neutrophil extracellular trap formation, known as NETosis, is abnormally active in cirrhosis associated inflammation. Activated neutrophils release NETs composed of DNA scaffolds and antimicrobial proteins. Although NETs can trap pathogens, their excessive formation damages vascular endothelium, promotes microthrombus formation, and exacerbates liver sinusoidal endothelial injury (Wu et al., 2021; Ortega-Ribera et al., 2026).

At the level of T cell immunity, a shift in the balance between T helper 17 (Th17) cells and Treg cells toward Th17 dominance is another characteristic change. LPS and cytokines such as IL-6 promote the differentiation of naive T cells into Th17 cells, which produce IL-17A. This recruits neutrophils and exacerbates tissue injury. In contrast, the proportion and function of Treg cells are relatively insufficient to effectively suppress the excessive immune response (Ni et al., 2017; Tan et al., 2021). This paradoxical state of “proinflammatory dominance coexisting with immunosuppression” is a unique feature of the immunopathology of decompensated HBV-related cirrhosis. It implies that any immunomodulatory intervention must be carefully balanced.

In summary, gut-liver axis dysregulation in decompensated HBV-related cirrhosis is not an isolated abnormality of a single factor but rather a systemic disease state characterized by multiple positive feedback loops among five major components: microbial community structure, intestinal barrier, bacterial translocation, metabolic networks, and immune responses. The abnormality at each node both originates from upstream drivers and reinforces downstream pathology, ultimately forming a self perpetuating vicious spiral.

## 3 Current microbiome-targeted interventions and their limitations

Before FMT emerged as a holistic restorative strategy, microbiome-targeted interventions in patients with decompensated cirrhosis had been explored for many years. However, none of these approaches has yet evolved into a well-established, standard therapy capable of reversing gut-liver axis dysregulation. These interventions primarily include probiotic and prebiotic supplementation, gut-decontaminating antibiotic therapy, as well as dietary modulation and foundational work on microbiota preparation techniques, which have received increasing attention in recent years. A critical appraisal of the efficacy and limitations of these modalities helps to explain why a single intervention is unlikely to achieve structural restoration of the gut-liver axis (Figure 2).

**Figure 2:**
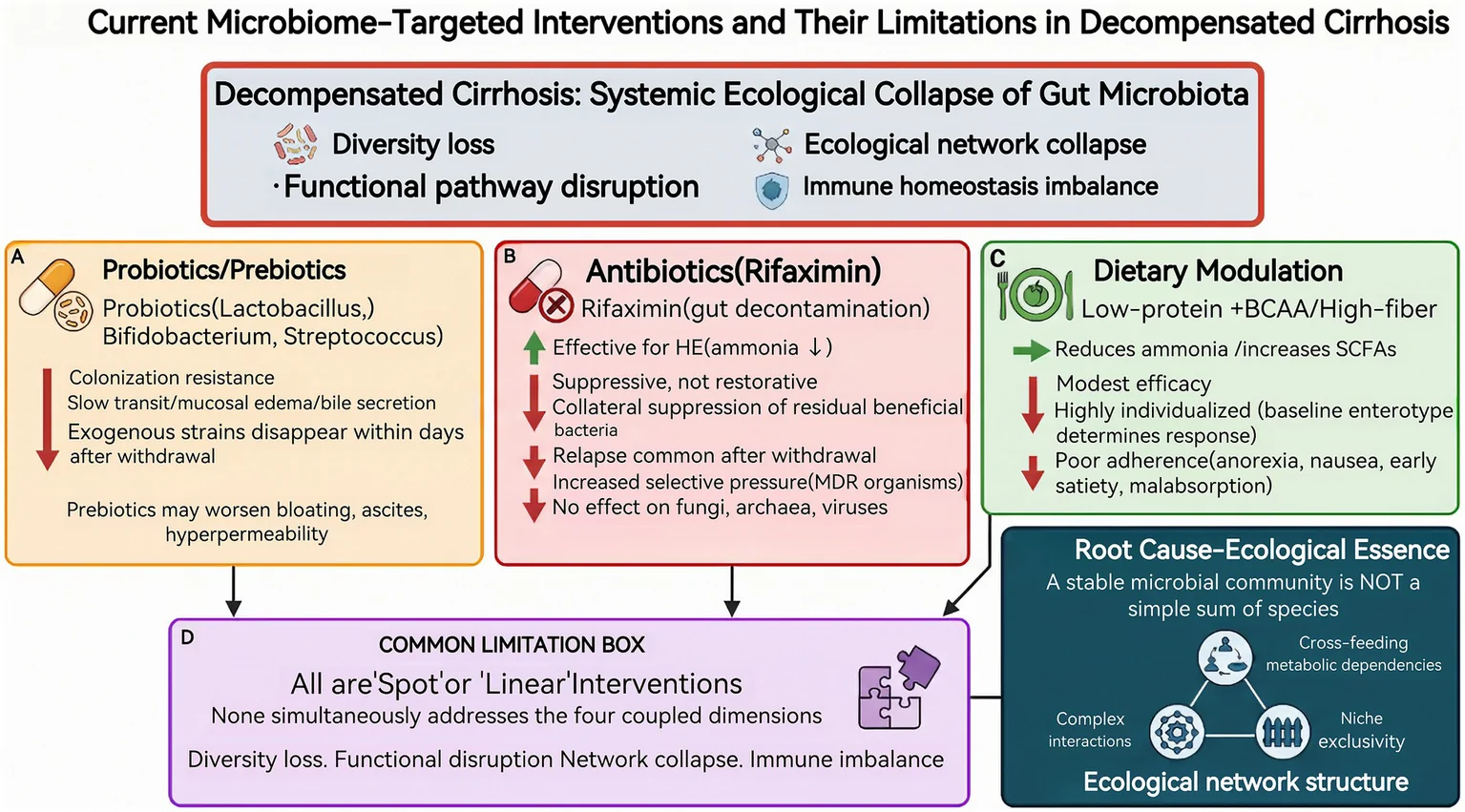
Current microbiome-targeted interventions in decompensated cirrhosis and their common limitations. Decompensated cirrhosis is characterized by systemic ecological collapse of the gut microbiota across four coupled dimensions: diversity loss, functional pathway disruption, ecological network collapse, and immune homeostasis imbalance. **(A)** Probiotics/Prebiotics: Exogenous strains face formidable colonization resistance due to slow intestinal transit, mucosal congestion/edema, reduced bile secretion, and frequent antibiotic use. Even during continuous administration, engraftment is limited, and exogenous strains disappear within days to weeks after withdrawal. Prebiotics may paradoxically exacerbate bloating, ascites, and intestinal hyperpermeability in some patients. **(B)** Antibiotics (rifaximin): Rifaximin effectively lowers blood ammonia and reduces HE episodes by suppressing urease-producing Gram-negative bacilli. However, its effect is suppressive rather than restorative. It also suppresses residual beneficial bacteria, relapse after withdrawal is common, and long-term use increases selective pressure for multidrug-resistant organisms. Moreover, rifaximin has no effect on fungi, archaea, or viruses. **(C)** Dietary modulation: Low-protein diets with BCAA supplementation reduce gut ammonia production, and high-fiber diets increase SCFAs. However, efficacy is modest and highly individualized because baseline enterotype determines metabolite profiles. Adherence is poor in decompensated patients who frequently experience anorexia, nausea, early satiety, and malabsorption. **(D)** All three interventions are “spot” or “linear” in nature. None simultaneously addresses the four coupled dimensions of dysbiosis. The fundamental reason for failure is the neglect of the ecological essence of the gut microbiota. A stable microbial community is not a simple sum of species but rather a network structure characterized by complex interactions, cross-feeding metabolic dependencies, and niche exclusivity. Restoration of the gut-liver axis requires a holistic strategy that targets the entire ecological network rather than isolated components. (BCAA, branched-chain amino acid; HE, hepatic encephalopathy; SCFAs, short-chain fatty acids).

### 3.1 Probiotics and prebiotics

Probiotics and prebiotics are the most widely used microbiome-modulating products in current clinical practice. However, the quality of evidence supporting their use in decompensated cirrhosis remains consistently low. Most probiotic studies employ a limited number of strains from *Lactobacillus*, *Bifidobacterium*, or *Streptococcus* species. Although these strains exert certain immunomodulatory and barrier-protective effects in healthy individuals, when transplanted into the profoundly disturbed intestinal ecosystem of decompensated cirrhosis, they face formidable colonization resistance. Patients with decompensated cirrhosis typically exhibit slowed intestinal transit, mucosal congestion and edema due to portal hypertensive enteropathy, reduced bile acid secretion, and frequent antibiotic use. Collectively, these factors shape a microenvironment that is highly unfavorable to the engraftment of exogenous strains (Costa et al., 2025; Verma et al., 2025). Most studies have used the detection of exogenous strains in feces as a marker of successful colonization. The results show that even during continuous administration, the detection rate is rather limited, and once supplementation ceases, exogenous strains typically disappear completely within days to weeks (Mohamad Nor et al., 2021; Chong et al., 2021).

The rationale behind prebiotics is to selectively promote the growth of endogenous beneficial bacteria by providing specific carbohydrates. This strategy is reasonable in healthy individuals or those with mild gut dysbiosis. However, in patients with decompensated cirrhosis, key functional bacterial groups such as butyrate-producing bacteria and secondary bile acid-producing bacteria are already severely depleted or even extinct. A more pragmatic concern is that patients with cirrhosis often have small intestinal bacterial overgrowth. As fermentable substrates, prebiotics may paradoxically exacerbate abdominal bloating and ascites in some patients, thereby limiting their clinical application to a certain extent (Tapper et al., 2023).

### 3.2 Antibiotics

Antibiotic gut decontamination, particularly the use of rifaximin for the prevention and treatment of HE, represents one of the few microbiome-targeted interventions supported by high-level evidence in the management of cirrhotic complications. Rifaximin more effectively maintained remission of hepatic encephalopathy and significantly reduced the risk of hospitalization due to hepatic encephalopathy (Bass et al., 2010). In addition, by suppressing urease- and ammonia-producing Gram-negative bacilli in the gut, rifaximin effectively lowers blood ammonia levels and thereby reduces both the frequency and severity of HE episodes. This strategy has been incorporated into multiple international guidelines (Pose et al., 2025; Singal et al., 2025).

However, the limitations of antibiotic intervention are equally evident. First, the therapeutic effect of rifaximin is essentially “suppressive” rather than “restorative.” While it eliminates pathogenic bacteria, it also suppresses residual beneficial bacteria, and long-term use further compresses the already reduced gut microbial diversity. Second, relapse after antibiotic discontinuation is extremely common, indicating that rifaximin does not truly address the deep-seated drivers of microbial dysbiosis. More critically, patients with cirrhosis are often at high risk for multidrug-resistant organisms. Repeated or prolonged use of any antibiotic increases selective pressure for resistance among opportunistic pathogens such as *Enterococcus*. Furthermore, antibiotics act exclusively on bacteria and have no effect on other gut microbial components, including fungi, archaea, and viruses. Emerging evidence has increasingly shown that gut dysbiosis in cirrhosis is a pan-microbiome disturbance, and that overgrowth of opportunistic fungi such as *Candida* species also contributes to hepatic inflammation and barrier injury (Hsu and Schnabl, 2023; Smets et al., 2025).

### 3.3 Dietary modulation

Beyond probiotics and antibiotics, dietary modulation has gained increasing attention in recent years as a natural and relatively safe intervention. A low-protein diet combined with branched-chain amino acid supplementation is a classic strategy in the nutritional management of patients with cirrhosis, aimed at reducing gut ammonia production and counteracting muscle wasting. A high-fiber diet is thought to enhance intestinal barrier function by increasing SCFAs production (Amodio et al., 2013). However, the central issue with dietary modulation lies in its modest efficacy and high degree of individualization. Gut microbial metabolism of dietary components is strongly influenced by the patient’s residual microbiota composition. Different baseline enterotypes may yield entirely different metabolite profiles in response to the same dietary fiber, meaning that dietary interventions are difficult to standardize and scale. More critically, against a backdrop of common symptoms in decompensated patients, including anorexia, nausea, early satiety, and malabsorption, achieving and maintaining an ideal dietary pattern is itself a clinical challenge.

Reviewing the common limitations of the various interventions described above reveals a core logic. The fundamental reason why a single intervention cannot completely restore the gut-liver axis is that gut dysbiosis in decompensated cirrhosis is a multidimensional, multilevel systemic collapse, rather than an isolated defect in a single bacterial species or a single metabolic pathway. Probiotics attempt to supplement one or two bacterial species; antibiotics try to suppress a few pathogenic groups; prebiotics aim to nourish residual beneficial bacteria; and dietary modulation seeks to alter the supply of metabolic substrates. However, all of these interventions are “spot” or “linear” in nature. None simultaneously addresses the four mutually coupled dimensions of diversity loss, functional pathway disruption, ecological network collapse, and immune homeostasis imbalance. More fundamentally, these interventions almost all neglect the ecological essence of the gut microbiota: a stable microbial community is not a simple sum of species, but rather a network structure characterized by complex interactions, cross-feeding metabolic dependencies, and niche exclusivity.

## 4 FMT in decompensated cirrhosis: rationale and clinical research progress

### 4.1 Rationale for FMT intervention

The defining feature that distinguishes FMT from traditional microbiome-targeted interventions such as probiotics or prebiotics is that FMT delivers an intact microbial community with ecological self-organization capacity, rather than single or a few microbial strains. Therefore, the therapeutic rationale for FMT in decompensated cirrhosis should be defined as “restoration” rather than “supplementation,” the former refers to the holistic recovery of ecosystem structure and function, whereas the latter can only achieve partial, transient compositional corrections. Based on the multistep pathological characteristics of gut-liver axis dysregulation, the theoretical targets of FMT-mediated restoration of this axis can be summarized into five interrelated functional modules.

At the level of ecological restoration, the core goal of FMT is to reverse the “pathogenic shift” of the gut microbiota, characterized by overgrowth of Enterobacteriaceae, in patients with decompensated cirrhosis. Strictly anaerobic commensals from the donor feces, predominantly Firmicutes and Bacteroidetes, occupy intestinal mucosal adhesion sites and nutritional niches through competitive exclusion. Simultaneously, they restore the metabolic niche blocking effect mediated by SCFAs. Butyrate inhibits nitrate reductase expression by activating PPARγ, thereby reducing intraluminal nitrate concentrations and depriving Enterobacteriaceae of their respiratory advantage in an anaerobic environment (Bajaj et al., 2014; Shao et al., 2018). More critically, the functional redundancy inherent in the healthy donor microbiota confers buffering capacity against the loss of individual bacterial species. At the level of barrier repair, donor-derived butyrate-producing bacteria upregulate tight junction protein expression and activate the HIF-1α-MUC2 axis, thereby enhancing mucus layer thickness and viscoelasticity (Peng et al., 2009). Concurrently, restoration of secondary bile acids induces antimicrobial peptide secretion via the FXR pathway, establishing a chemical barrier that eliminates bacteria breaching the mucus layer (Lefebvre et al., 2009; Gadaleta et al., 2011; Wang et al., 2011). The positive feedback loop between barrier repair and ecological restoration constitutes the kinetic basis for sustained amplification of the FMT effect.

At the level of metabolic reversal, FMT restores 7α/β-dehydroxylase activity of bacterial groups including Clostridium clusters XIVa and IV, thereby reestablishing the conversion pathway from primary to secondary bile acids. The latter inhibits NLRP3 inflammasome activation in the liver via the TGR5 receptor (Ridlon et al., 2006; Chiang, 2009; Jia et al., 2018; Lau et al., 2025). The synergistic restoration of butyrate, acetate, and propionate production rebalances hepatic homeostasis through both energy metabolism and inflammatory regulation. Furthermore, repair of the tryptophan-indole-AhR-IL-22 signaling axis further integrates metabolic and immune modulation (Fujigaki et al., 2001; Laurans et al., 2018). At the level of immune regulation, FMT reduces the net translocation of gut-derived LPS, thereby relieving Kupffer cells from persistent TLR4 stimulation and reversing their immune paralysis phenotype (Tacke, 2017; Peiseler et al., 2023; Horn and Tacke, 2024). Butyrate and secondary bile acids synergistically promote the differentiation of CD4^+^ T cells toward the Treg lineage while inhibiting Th17 polarization, thereby reshaping the Th17/Treg balance (Ni et al., 2017; Tan et al., 2021).

At the level of ammonia load reduction, FMT introduces *Lactobacillus* and *Bifidobacterium* species with high ammonia-assimilating capacity, redirecting ammonia from a metabolic waste product to a nitrogen source. Simultaneously, restoration of SCFA-induced intestinal luminal acidification promotes the conversion of NH₃ to poorly absorbable NH₄^+^ (Frieg et al., 2021; Gallego-Durán et al., 2024; Ziki and Colnot, 2024).

### 4.2 Clinical research progress

The earliest studies in this area were case series and open-label trials, primarily small exploratory studies. These studies consistently reported short-term improvements in Child-Pugh and MELD scores, a marked reduction in the annual incidence of HE, and fewer infection-related hospital events such as SBP following FMT intervention (Kao et al., 2016; Mehta et al., 2018). However, due to the lack of contemporaneous controls and the presence of significant selection bias, the generalizability of these findings is limited. Several small-sample randomized controlled trials (RCTs) have been published in recent years, mainly focusing on secondary prevention of HE. The THEMATIC trial by Bajaj and colleagues represents the highest-quality design in this field to date, a double-blind, placebo-controlled study. The results showed that the 180-day HE recurrence rate in the FMT group was significantly lower than that in the placebo group (9% vs. 40%), and the efficacy remained stable regardless of administration route or donor type (Bajaj et al., 2019). In addition, Sharma et al., in an open-label RCT of alcohol-associated ACLF, found that 28-day and 90-day survival rates were significantly better in the FMT group than in the standard-of-care group. Although this signal comes from a non-HBV etiology, it provides preliminary evidence for the safety of FMT in critically ill patients with liver failure (Sharma et al., 2022).

However, the limitations of existing RCTs should not be overlooked. The substantial heterogeneity in donor screening criteria, fecal preparation procedures, and administration protocols makes direct comparisons between studies difficult. Follow-up durations are generally less than 6 months, precluding assessment of the long-term impact of FMT on hard clinical endpoints such as death and liver transplantation. More critically, most studies have focused on cirrhosis of alcohol-associated or metabolic dysfunction-associated steatotic liver disease (MASLD) etiology, with patients with HBV either underrepresented or explicitly excluded. Analysis specific to the subgroup of decompensated HBV-related cirrhosis is entirely lacking.

Regarding safety, available evidence generally supports an acceptable safety profile for FMT in patients with cirrhosis. The THEMATIC trial reported no FMT-related serious adverse events. Nevertheless, theoretical concerns remain regarding bacteremia, transmission of multidrug-resistant organisms, and procedure-related complications. Of particular note, patients with cirrhosis are predisposed to immune paralysis and bacterial translocation. Should FMT-related infection occur, the consequences could be more severe than in other populations.

In response to the unique host environment of decompensated cirrhosis, several specialized FMT strategies have been developed in recent years to enhance safety and engraftment efficiency. Washed microbiota transplantation, which removes pyrogens, toxic metabolites, and potential pathogen fragments from donor feces through centrifugation and resuspension steps, reduces the risk of post-infusion fever and endotoxemia and has demonstrated better tolerability in patients with cirrhosis and ascites. The advent of frozen capsule formulations has made it possible to perform FMT without invasive procedures such as colonoscopy or nasojejunal tube placement, a particularly important advance for patients with cirrhosis who may have coagulopathy or esophageal varices. The concept of anaerobically processed microbiota stems from the recognition that strictly anaerobic bacteria die in large numbers upon exposure to oxygen. Microbiota suspensions prepared under strict anaerobic conditions retain a higher proportion of butyrate-producing bacteria, and preliminary studies suggest they are superior to conventionally prepared formulations in improving blood ammonia levels and cognitive function.

Refinement of donor selection strategies represents another key advance. Researchers are no longer relying solely on routine stool testing and pathogen screening. Instead, they are beginning to use metagenomic data to select so-called “super donors” characterized by high butyrate-producing capacity, low urease activity, and an anti-inflammatory phenotype. This precision matching approach holds promise for addressing the problem of heterogeneous responses to FMT commonly observed in patients with decompensated cirrhosis.

In summary, clinical research on FMT in cirrhosis, particularly for HE, has progressed from case series to phase II RCTs. However, evidence specific to HBV-related decompensated cirrhosis is almost entirely lacking. Rigorously designed, donor-standardized, adequately followed multicenter RCTs are urgently needed.

## 5 Challenges and bottlenecks

Although FMT has demonstrated unique theoretical advantages and preliminary clinical promise in decompensated cirrhosis, this field still faces a series of pressing challenges and controversies. These range from donor selection to microbiota formulation, from HBV-specific issues to the impact of cirrhosis on FMT engraftment, and from the lack of consensus on outcome measures to concerns about long-term safety. These challenges are not merely technical obstacles; they bear directly on whether FMT can transition from exploratory research to standardized clinical application.

### 5.1 Donor selection and formulation heterogeneity

The lack of consistency in donor selection and microbiota formulation is the most prominent source of heterogeneity in current FMT research. Regarding whether donor feces should be freshly prepared or cryopreserved, available evidence supports that cryopreserved formulations are non-inferior to fresh feces, and cryopreservation facilitates standardized quality control and long-term storage. However, differences in cryoprotectants, cooling rates, and storage durations across studies make it difficult to assess variability in formulation activity. Regarding administration routes, nasoenteric tubes, colonoscopic enemas, and oral capsules each have advantages and disadvantages. Nasoenteric tubes can reach the duodenum and allow repeated dosing, but the procedure is invasive and requires hospitalization. Colonoscopy permits simultaneous evaluation of the intestinal mucosa, but the risks of sedation and perforation must be carefully weighed in patients with cirrhosis. Capsule formulations are the most acceptable to patients and can be used in an outpatient setting, but concerns remain regarding gastric acid degradation, absorption in the upper gastrointestinal tract, and uncertain engraftment efficiency. The THEMATIC trial suggested that efficacy did not differ significantly across routes, but this conclusion requires validation in larger samples.

A deeper controversy lies in the choice between “whole microbiota” and “defined microbial consortia.” Whole microbiota retains all microbial and metabolite components of donor feces. In theory, best mimics the functional redundancy of a healthy ecosystem. However, its composition is complex, its mechanisms are poorly defined, and it carries the risk of transmitting unknown pathogens. Defined microbial consortia are more controllable, but single or few strains are unlikely to achieve durable engraftment in the severely dysbiotic cirrhotic gut and may underestimate the importance of synergistic microbial metabolism.

### 5.2 HBV-specific considerations

HBV-specific issues have long been neglected in this field. The vast majority of current FMT clinical studies have focused on cirrhosis of alcohol-associated or MASLD etiology, with HBV patients either underrepresented or explicitly excluded. This raises two key questions. First, in patients with decompensated HBV-related cirrhosis who are receiving effective antiviral therapy with persistently undetectable HBV DNA, does an independent gut–liver axis disturbance still exist? Even when viral replication is completely suppressed, established intestinal barrier injury, dysbiosis, and metabolic abnormalities do not reverse spontaneously. This suggests that gut–liver axis dysregulation in HBV-related cirrhosis has the characteristics of a “post-viral sequela.” Second, does FMT affect the pharmacokinetics of nucleos(t)ide analogs (NAs)? NAs are primarily metabolized via the kidneys or liver. Theoretically, FMT-induced changes in the gut microbiota could affect the expression of drug transporters, thereby altering NA absorption and excretion. No study has directly addressed this question, but given that patients with HBV require lifelong NA therapy, any potential drug–microbiota interaction warrants rigorous evaluation.

### 5.3 Cirrhosis-related barriers to FMT engraftment

The pathophysiological characteristics of cirrhosis itself pose unique obstacles to FMT engraftment efficiency. Patients with decompensated cirrhosis commonly exhibit delayed gastric emptying, abnormal small intestinal motility, and prolonged colonic transit time. These motility disorders can affect the distribution and retention of donor microbiota in the gut. More critically, the bile acid pool is markedly altered in patients with cirrhosis, characterized by increased primary bile acids and decreased secondary bile acids. Secondary bile acids are essential signaling molecules for the growth of certain commensal bacteria. This abnormality of the “bile acid niche” makes it difficult for secondary bile acid-dependent bacterial groups from healthy donors to establish populations in the cirrhotic gut. Furthermore, the vast majority of patients with cirrhosis have been on long-term rifaximin or lactulose before enrollment in FMT studies. Standardized protocols for washout periods are lacking. Too short a washout may allow residual drugs to suppress donor microbiota, whereas too long a washout exposes patients to the risk of HE recurrence. No study has systematically compared the impact of different washout durations on FMT engraftment efficiency, representing a critical bottleneck impeding optimization of clinical protocols.

### 5.4 Lack of consensus on outcome measures

The lack of consensus on outcome measures is another core issue constraining evidence synthesis in this field. Existing studies have employed diverse endpoints, including biochemical markers (blood ammonia, bilirubin, albumin), dynamic scores (MELD, Child-Pugh), measures of microbial restoration (alpha diversity, abundance of specific taxa), hard clinical endpoints (death, liver transplantation, ACLF), and patient-reported outcomes (quality of life, HE recurrence rate). In the absence of a unified core outcome set, cross-study comparisons are extremely difficult, and the conclusions of meta-analyses are consequently limited. More critically, the association between surrogate endpoints (e.g., reduction in blood ammonia) and hard clinical endpoints is not robust. A patient with decreased blood ammonia may not necessarily be spared from ACLF or death, and restoration of microbial diversity does not necessarily equate to functional reconstitution. Therefore, future clinical trials urgently need to establish a validated, hierarchical outcome framework that includes both short-term surrogate endpoints and long-term hard clinical endpoints.

### 5.5 Long-term safety concerns

Long-term safety concerns represent the greatest unanswered question hanging over this field. Short-term safety data are generally reassuring, but long-term follow-up studies are virtually absent. A recurring but unresolved concern is whether specific microbial metabolites play a “double-edged sword” role in the transition from cirrhosis to hepatocellular carcinoma. In addition, the long-term risks of potential oncogenic viruses and resistance genes carried in donor feces require evaluation through prospective cohort studies.

In summary, the clinical application of FMT in decompensated HBV-related cirrhosis must overcome multiple barriers, including donor standardization, validation of HBV-specific considerations, elucidation of engraftment obstacles, harmonization of outcome measures, and confirmation of long-term safety. These challenges do not negate the potential of FMT; rather, they underscore the urgent need for rigorously designed, adequately followed, multicenter collaborative studies to answer the core clinical question: for whom, when, how, and with what type of FMT?

### 5.6 Regulatory and cost barriers

In addition to the scientific and technical challenges outlined above, the clinical translation of FMT in cirrhosis faces considerable regulatory and cost-related obstacles. This issue, while seldom discussed in the current literature, represents a major bottleneck constraining the transition of FMT from research to clinical practice.

#### 5.6.1 Fragmented and ambiguous regulatory frameworks

To date, no global consensus exists on the regulatory classification of FMT across major countries and regions. In the United States, the Food and Drug Administration (FDA) regulates FMT as both a “biological product” and a “drug,” requiring an Investigational New Drug (IND) application for clinical trials. Nevertheless, the FDA has exercised enforcement discretion for FMT in the treatment of *Clostridioides difficile* infection (CDI), temporarily waiving the IND requirement for this specific indication. However, it remains unclear whether this discretion applies to other indications, including cirrhosis. In Europe, the European Medicines Agency (EMA) classifies FMT as an “advanced therapy medicinal product” (ATMP) subject to a centralised authorisation process, yet considerable variation exists in its practical implementation across member states. In China, neither the National Health Commission nor the National Medical Products Administration has issued dedicated regulations for FMT. Currently, FMT is managed as a “medical technology” rather than being subjected to the pharmaceutical approval pathway. This regulatory ambiguity has given rise to a series of downstream consequences, including inconsistent donor screening criteria, the absence of Good Manufacturing Practice (GMP) grade quality control requirements for faecal slurry preparation, substantial inter-institutional variability in manufacturing protocols, and inadequate adverse event reporting systems. For patients with decompensated cirrhosis, a population inherently predisposed to immune dysfunction and bacterial translocation, the lack of unified regulatory oversight raises serious concerns regarding patient safety.

#### 5.6.2 High costs and lack of reimbursement coverage

The clinical uptake of FMT is further constrained by significant economic barriers. From a cost perspective, the expenses associated with FMT mainly include donor screening and matching (encompassing blood tests, stool pathogen screening, multidrug-resistant organism testing, and optional human leukocyte antigen typing or metagenomic profiling), faecal slurry preparation and storage (processing under strict anaerobic conditions, cryopreservation, and quality control), and the delivery procedure itself (nasojejunal tube placement, colonoscopy, or capsule-based administration). In the United States, the estimated cost of a single FMT session ranges from USD 1,500 to 3,500. In China, a single colonoscopy-delivered FMT procedure costs approximately RMB 8,000 to 20,000, with capsule formulations being even more expensive. For patients with decompensated cirrhosis who may require repeated FMT sessions to maintain therapeutic benefit, the cumulative cost can be prohibitive. Critically, no healthcare system worldwide currently provides routine insurance coverage for FMT in the context of cirrhosis-related complications. In the United States, FMT has only obtained limited reimbursement for recurrent CDI. In China, FMT remains entirely out-of-pocket and has not yet been included in the National Reimbursement Drug List or the catalogue of reimbursable medical procedures. Given that cirrhosis is a chronically progressive disease, patients already bear substantial medical expenditures for long-term antiviral agents, diuretics, endoscopic therapies, and other interventions. The added financial burden of FMT would further strain their resources, severely compromising both the accessibility and equity of this therapeutic modality.

#### 5.6.3 A self-reinforcing vicious cycle between regulatory and cost barriers

Notably, these two sets of barriers are mutually reinforcing. The lack of clear regulatory standards discourages industrial investment in the development of standardised, scalable FMT products, as the absence of a well-defined approval pathway renders the return on investment highly unpredictable. In turn, the dearth of commercially available, GMP-compliant, mass-producible products perpetuates high costs and heterogeneous quality. These issues, namely excessive costs and variable product quality, further impede the execution of large-scale randomised controlled trials. The consequent lack of robust clinical evidence, in turn, delays regulatory clarity and the expansion of insurance coverage. This vicious cycle lies at the heart of the slow progress in translating FMT into clinical practice for cirrhosis.

## 6 Future directions and clinical translation pathways

Looking ahead, research on FMT in decompensated HBV-related cirrhosis urgently needs to transition from the preliminary validation phase of “whether it works” to a phase of precision and mechanistic understanding that addresses “for whom it works, how to optimize it, why it works, and whether it is safe.” This transition requires the coordinated advancement of multidimensional research designs, with the establishment of stratified FMT strategies as the highest priority.

### 6.1 Stratified FMT strategies

Current research treats all patients with decompensated cirrhosis as a homogeneous population, yet substantial differences exist among patients in terms of enterotype, immune status, and hepatic functional reserve. An enterotype-based strategy implies that patients with *Bacteroides*-dominant, *Prevotella*-dominant, and *Ruminococcus*-dominant enterotypes may respond completely differently to FMT. Gut enterotypes are typically defined after 16S rRNA sequencing by the relative abundance of dominant genera at the genus level; for example, *Bacteroides* >30% or *Prevotella* >15% can be assigned to the corresponding enterotype. However, these thresholds vary with the sequencing region and clustering method, so they must be standardized in advance within each study. Immune status is assessed using a composite index, such as an immune index that integrates IgG, complement, CD4^+^/CD8^+^ T cells, and routine inflammatory markers including CRP, IL-6, and SII, with 50–80 as the reference range for immune homeostasis. Child-Pugh classification is based on five items: bilirubin, albumin, prothrombin time, ascites, and HE, with scores of 5–6, 7–9, and 10–15 corresponding to grades A, B, and C, respectively. Future validation should incorporate these strata into prospective cohorts to verify their independent predictive value for treatment response or survival. For example, patients with a *Prevotella*-dominant enterotype typically have a higher capacity for dietary fiber fermentation and may be more receptive to engraftment of butyrate-producing bacteria from the donor microbiota. Stratification based on immune status focuses on baseline inflammatory levels. Using parameters such as the Th17/Treg ratio and plasma concentrations of IL-6 and TNF-α, the therapeutic targets of FMT differ between patients with immune hyperactivation and those with immune paralysis. For the Th17/Treg ratio or IL-6 level, the optimal thresholds should be determined primarily from prospective cohort data using ROC curve analysis, with the cutoff defined according to the specific endpoint of interest, such as FMT response or infection risk. Immune hyperactivation refers to an excessive proinflammatory response, characterized by an elevated Th17/Treg ratio and massive release of proinflammatory cytokines such as IL-6 and TNF-α, which exacerbates liver injury. Immune paralysis, by contrast, refers to a state in which the immune system shifts toward suppression after prolonged inflammation, with reduced proinflammatory capacity and impaired antigen presentation; although inflammatory markers may not be markedly elevated, the ability to combat infection and clear pathogens is diminished. These two conditions represent opposite directions of immune dysregulation and therefore require different FMT intervention targets. The former requires priority suppression of inflammation, whereas the latter needs restoration of Kupffer cell function. The Child-Pugh classification, as the simplest tool for stratifying hepatic function, can be directly used to define the optimal window for FMT intervention. Patients with Child-Pugh B cirrhosis may have the greatest “plasticity,” whereas those with Child-Pugh C cirrhosis may have reached an irreversible stage of intestinal barrier injury, potentially resulting in extremely low FMT engraftment efficiency. Establishing an integrated stratification model incorporating these three dimensions and prospectively validating its predictive value for FMT efficacy is a core task in advancing personalized FMT.

### 6.2 Engineered and synthetic microbial consortia

At the formulation level, engineered or synthetic microbial consortia represent a next-generation strategy beyond traditional FMT. Although whole fecal microbiota has the advantage of functional redundancy, its compositional complexity poses practical problems, including difficulty in donor screening, challenges in standardizing quality control, and the risk of transmitting potential pathogens. The core logic of engineered microbial consortia is to “keep the essence while discarding the dross,” etaining the core functional modules of a healthy microbiota while eliminating unnecessary or potentially harmful components. Specifically, synthetic consortia composed of selected bacterial species can be constructed and endowed with additional therapeutic functions through genetic engineering. Such rationally designed synthetic consortia are not only more controllable in terms of safety but also avoid batch-to-batch variability arising from donor individual differences. However, whether synthetic consortia can achieve engraftment efficiency comparable to that of whole microbiota in the hostile environment of the cirrhotic gut, and whether the cross-feeding relationships among multiple species established *in vitro* can be recapitulated *in vivo*, require in-depth engineering validation.

Engineered synthetic consortia offer clear advantages: their composition is well defined, safety is more controllable, and batch-to-batch inconsistency caused by donor variability can be avoided. However, whether they can reproduce the functional redundancy of an intact microbiota remains uncertain. Whole fecal microbiota possesses functional redundancy, allowing it to maintain functional stability under perturbation through shared metabolic pathways across multiple species. In contrast, synthetic consortia retain only a limited number of strains, so if a key strain fails to colonize, the resulting functional loss may not be compensated for.

As genetically modified organisms, engineered consortia face multiple regulatory and safety challenges in clinical application. The United States FDA regulates them as biological products, requiring an IND submission and GMP compliance, yet dedicated guidance for live biotherapeutic products remains incomplete. Core safety concerns include the risk of environmental escape and horizontal gene transfer, *in vivo* genetic stability, and the ecological impact of long-term colonization. Current biocontainment strategies, such as auxotrophy and kill switches, still require standardized validation.

### 6.3 Combination therapeutic strategies

The design of combination therapeutic strategies represents another key pathway to enhance the clinical benefit of FMT. FMT should not be viewed as an isolated intervention but rather as a core component of a comprehensive treatment regimen, working synergistically with other therapeutic modalities. Synergy with antiviral therapy is the most fundamental consideration. Stable viral suppression is a prerequisite for FMT to exert its effects, but whether FMT can reduce the long-term dosage of NAs or improve their renal safety warrants exploration. Combination with rifaximin requires careful temporal design. Rifaximin can serve as a preoperative “gut decolonization” measure to reduce competition from the recipient’s endogenous pathogenic bacteria against donor microbiota engraftment. However, residual antibiotics can also inhibit donor microbiota. The ideal washout window may be only 2 to 3 days, a hypothesis that needs validation through pharmacokinetic and pharmacodynamic studies. Combination with albumin focuses on improving systemic circulatory function and endotoxin clearance in patients with cirrhosis. Long-term albumin infusion can reduce plasma LPS levels, potentially creating a more favorable immune microenvironment for FMT engraftment. The synergistic effects of these combination strategies need to be systematically evaluated in factorial design clinical trials.

### 6.4 Long-term follow-up and multi-omics integration

Long-term follow-up and multi-omics integration constitute the technical foundation for revealing the mechanisms of FMT-mediated “restoration” and predicting long-term outcomes. After patients with decompensated cirrhosis receive FMT, microbiota reconstitution is not an instantaneous event but rather a dynamic evolutionary process that may go through three stages: engraftment, expansion, and stabilization. Traditional endpoint analyses that measure microbial diversity at a single time point cannot capture this dynamic trajectory. Therefore, future studies should implement multi-time-point sampling protocols extending for at least 12 months after FMT, with concurrent performance of metagenomic sequencing (to track microbial community structure and functional genes), metabolomics (to detect bile acids, short-chain fatty acids, trimethylamine N-oxide, and tryptophan metabolites), and immune profiling (plasma cytokines, monocyte HLA-DR, Treg/Th17 ratio). By integrating these multi-omics data, a dynamic network model of “microbiota-metabolism-immune” reconstitution can be constructed, identifying key nodes predictive of successful engraftment and clinical response, thereby providing real-time guidance for clinical decision-making. Furthermore, long-term follow-up must include surveillance for the development of HCC. Because cirrhosis itself is a high-risk condition for HCC, any new intervention must rule out long-term carcinogenic risk. This requires the establishment of prospective registry systems to track FMT recipients for at least 5 to 10 years.

Ultimately, the effective advancement of all the above directions depends on rigorously designed, multicenter, double-blind, outcome-assessor-blinded RCTs. Such studies need to pay particular attention to several key design elements. First, specific subgroups of decompensated cirrhosis (HBV-related, Child-Pugh B or C, with or without prior history of HE) must be predefined to ensure the pertinence of the conclusions. Second, central randomization with allocation concealment should be employed to avoid selection bias. Third, donor feces should be sourced from a strictly screened, standardized donor bank, with uniform preparation and storage protocols. Finally, the primary outcomes should be hard clinical endpoints (e.g., time to first HE recurrence, incidence of acute-on-chronic liver failure, 180-day mortality) rather than surrogate markers, and a safety monitoring committee should be established to regularly review serious adverse events.

## 7 Conclusion

Gut-liver axis dysregulation is one of the core driving mechanisms of disease progression in decompensated HBV-related cirrhosis. In essence, it is a multistep, positive-feedback pathological process encompassing ecological collapse of the gut microbiota, intestinal barrier failure, metabolic network remodeling, and immune response dysregulation. FMT, by virtue of its unique ability to deliver a complete, functionally active microbial ecosystem, holds the theoretical potential to interrupt this vicious cycle at its source and achieve systematic restoration of the gut–liver axis.

Existing preclinical and clinical studies have preliminarily demonstrated short-term efficacy of FMT in reducing blood ammonia levels, decreasing recurrence of HE, improving liver function scores, and lowering the incidence of infectious events, with a generally acceptable safety profile. However, the quality of the current evidence base remains markedly insufficient. Studies specifically targeting HBV-related decompensated cirrhosis are virtually absent. Most randomized controlled trials are limited by a lack of donor standardization, inadequate follow-up duration, and insufficient subgroup analyses. Long-term safety, particularly the potential impact on the risk of hepatocellular carcinoma development, remains undetermined.

Looking ahead, progress in this field urgently needs to transition from empirical exploration to a new phase characterized by precision, engineering, and standardization. This will require the establishment of stratified FMT strategies based on enterotype and immune status, the development of functionally enhanced synthetic microbial consortia, the design of combination regimens integrating FMT with antiviral agents, rifaximin, or albumin, and the conduct of long-term follow-up, multi-omics-integrated, multicenter double-blind randomized controlled trials. Only through these efforts can FMT be transformed from a theoretical “restorative potential” into a generalizable and reproducible new clinical dimension in the comprehensive management of decompensated HBV-related cirrhosis.

## Appendix

Given the narrative nature of this review, we did not perform a formal systematic review with meta-analysis according to PRISMA guidelines. Instead, we conducted a comprehensive and structured literature search in PubMed, Web of Science, and CNKI (China National Knowledge Infrastructure) databases, covering the period from January 2000 to June 2026. The search terms combined MeSH terms and free-text keywords, including “hepatitis B virus,” “HBV,” “cirrhosis,” “decompensated cirrhosis,” “gut-liver axis,” “gut microbiota,” “dysbiosis,” “intestinal barrier,” “endotoxemia,” “fecal microbiota transplantation,” “FMT,” “hepatic encephalopathy,” “spontaneous bacterial peritonitis,” “bile acids,” “short-chain fatty acids,” and immune dysfunction.” We also manually screened the reference lists of retrieved articles and relevant review papers to identify additional eligible studies. Priority was given to high-quality evidence, including randomized controlled trials, large cohort studies, and mechanistic studies published in peer-reviewed journals.
